# Genotype sampling for deep-learning assisted experimental mapping of a combinatorially complete fitness landscape

**DOI:** 10.1093/bioinformatics/btae317

**Published:** 2024-05-15

**Authors:** Andreas Wagner

**Affiliations:** Department of Evolutionary Biology and Environmental Studies, University of Zurich, 8057 Zurich, Switzerland; Swiss Institute of Bioinformatics, Quartier Sorge-Batiment Genopode,1015 Lausanne, Switzerland; The Santa Fe Institute, Santa Fe, 87501 NM, United States

## Abstract

**Motivation:**

Experimental characterization of fitness landscapes, which map genotypes onto fitness, is important for both evolutionary biology and protein engineering. It faces a fundamental obstacle in the astronomical number of genotypes whose fitness needs to be measured for any one protein. Deep learning may help to predict the fitness of many genotypes from a smaller neural network training sample of genotypes with experimentally measured fitness. Here I use a recently published experimentally mapped fitness landscape of more than 260 000 protein genotypes to ask how such sampling is best performed.

**Results:**

I show that multilayer perceptrons, recurrent neural networks, convolutional networks, and transformers, can explain more than 90% of fitness variance in the data. In addition, 90% of this performance is reached with a training sample comprising merely ≈10^3^ sequences. Generalization to unseen test data is best when training data is sampled randomly and uniformly, or sampled to minimize the number of synonymous sequences. In contrast, sampling to maximize sequence diversity or codon usage bias reduces performance substantially. These observations hold for more than one network architecture. Simple sampling strategies may perform best when training deep learning neural networks to map fitness landscapes from experimental data.

**Availability and implementation:**

The fitness landscape data analyzed here is publicly available as described previously (Papkou *et al.* 2023). All code used to analyze this landscape is publicly available at https://github.com/andreas-wagner-uzh/fitness_landscape_sampling

## 1 Introduction

A fitness or adaptive landscapes is a high-dimensional analogue to a landscape in physical space. Each genotype of an organism or biomolecule corresponds to a spatial location, and the elevation at that location corresponds to fitness. Darwinian evolution can be viewed as an exploration of such a landscape that drives evolving populations towards high fitness peaks (Wright 1932). Characterizing the topography of a fitness landscape and identifying its highest peaks is important to both evolutionary biology and biomedical engineering.

The first experimental data on fitness landscapes became available only in the early 2000s, when experimental measurements of multiple mutations in the antibiotic resistance protein TEM-1 beta lactamase showed that few mutational paths to high antibiotic resistance are evolutionarily accessible, i.e. fitness-increasing for each mutational step (Weinreich *et al.* 2006). Since then, numerous experimental studies on the topography of adaptive landscapes have been published, some based on few genotypes (Hall *et al.* 2010, Chou *et al.* 2011, de Visser and Krug 2014, Mira *et al.* 2015, Palmer *et al.* 2015, Weinreich *et al.* 2018, Yang *et al.* 2019), others based on thousands (Olson *et al.* 2014, Li *et al.* 2016, Sarkisyan *et al.* 2016, Diss and Lehner, 2018, Domingo *et al.* 2018, Li and Zhang 2018, Bendixsen *et al.* 2019, Poelwijk *et al.* 2019, Pokusaeva *et al.* 2019). Some studies directly quantify organismal fitness conveyed by different genotypes (Hall *et al.* 2010, Chou *et al.* 2011, Palmer *et al.* 2015, Li *et al.* 2016, Domingo *et al.* 2018, Li and Zhang, 2018). Many others quantify molecular traits that can serve as a proxy for fitness, such as gene expression (Li *et al.* 2019), enzyme activity (Bendixsen *et al.* 2019, Yang *et al.* 2019), light emission by fluorescent proteins (Sarkisyan *et al.* 2016, Poelwijk *et al.* 2019, Zheng *et al.* 2019), strength of protein–protein interactions (Olson *et al.* 2014, Wu *et al.* 2016, Diss and Lehner, 2018), protein-RNA binding(Melamed *et al.* 2013), or protein-DNA binding (Aguilar-Rodriguez *et al.* 2017).

To study the topography of adaptive landscapes is challenging. First, current theoretical models cannot predict the fitness in such landscapes from genotype alone (Kauffman and Levin, 1987, de Visser and Krug 2014, Weinreich *et al.* 2018, Das and Krug 2022), because different base pairs interact in complex non-additive ways to determine a genotype’s fitness (Weinreich *et al.* 2005, Poelwijk *et al.* 2011, Weinreich *et al.* 2013, Domingo *et al.* 2018, Weinreich *et al.* 2018, Poelwijk *et al.* 2019, Yang *et al.* 2019). Second, adaptive landscapes have astronomical sizes. For example, even a small gene of 100 base pairs has 4^100^ = 1.6 × 10^60^ possible genotypes. The largest landscapes mapped to date have 10^5^–10^7^ characterized genotypes (Chou *et al.* 2011, Li *et al.* 2016, Sarkisyan *et al.* 2016, Domingo *et al.* 2018, Bendixsen *et al.* 2019, Pokusaeva *et al.* 2019, Vaishnav *et al.* 2022, Papkou *et al.* 2023).

Machine learning methods may help to map otherwise prohibitively large landscapes. This would involve a three-step process. First, experimentally measure the fitness of a manageable sample of DNA sequences from a landscape. Second, use the resulting data as training and validation data for a machine learning algorithm to predict the fitness of DNA sequences. Third, test these predictions by experimentally measuring the fitness of additional DNA sequences as a test set. If the algorithm generalizes well to the test set, it can be used to study the topography of the entire landscape. The emphasis on DNA sequences is important, because even synonymous DNA sequences, which encode the same amino acid sequence, can differ substantially in fitness (Cambray *et al.* 2018, Papkou *et al.* 2023). Unfortunately, most existing experimental landscape studies are not suited for this purpose. They either represent data on the amino acid level and do not quantify fitness for multiple synonymous DNA sequences, or they contain fitness data for too few DNA sequences to allow deep learning (Bershtein *et al.* 2015, Rodrigues *et al.* 2016, Diss and Lehner, 2018, Tamer *et al.* 2019, Lite *et al.* 2020, Huang *et al.* 2021, McCormick *et al.* 2021). Here I take advantage of a recently published dataset that overcomes these limitations (Papkou *et al.* 2023).

Machine learning in general, and deep learning in particular have proven highly successful in predicting biological phenomena (Romero *et al.* 2013, Alipanahi *et al.* 2015, Govindarajan *et al.* 2015, Riesselman *et al.* 2018, Alley *et al.* 2019, Flagel *et al.* 2019, Rao *et al.* 2019, Washburn *et al.* 2019, Adrion *et al.* 2020, Avsec *et al.* 2021, Fernandez-de-Cossio-Diaz *et al.* 2021, Xue *et al.* 2021, Tareen *et al.* 2022, Vaishnav *et al.* 2022, Zhou *et al.* 2022). For example, they can predict gene expression (Washburn *et al.* 2019, Vaishnav *et al.* 2022), protein structure and pathogenicity (Jumper *et al.* 2021, Cheng *et al.* 2023), protein stability (Pancotti *et al.* 2021, Blaabjerg *et al.* 2023), protein-nucleic acid binding (Avsec *et al.* 2021, Alipanahi *et al.* 2015), DNA methylation (Angermueller *et al.* 2017), mutational effects on proteins and RNA (Riesselman *et al.* 2018), ribosomal binding site activity (Höllerer *et al.* 2020), as well as recombination rates and selective sweeps (Flagel *et al.* 2019, Adrion *et al.* 2020, Xue *et al.* 2021).

Several studies have used machine learning to predict molecular phenotypes that can be correlated with fitness (Alley *et al.* 2019, Li *et al.* 2019, Xu *et al.* 2020, Wittmann *et al.* 2021, Tareen *et al.* 2022, Vaishnav *et al.* 2022). Some of them employ machine learning to reduce experimental effort in directed evolution experiments. Such experiments require labor-intensive screening of enzyme variants with desirable properties, such as a faster rate of enzymatic catalysis, to improve biotechnologically important enzymes (Li *et al.* 2019, Wu *et al.* 2019, Wittmann *et al.* 2021). The most pertinent existing work focuses on the small screening samples (10^1^–10^2^ enzyme variants) typical for directed evolution, and on machine learning methods different from deep learning (Li *et al.* 2019, Wu *et al.* 2019, Wittmann *et al.* 2021). It shows that a simple one-hot encoding or an encoding based on physicochemical amino acid properties can help to predict viable genotypes equally well or better than sophisticated encodings pre-learned on vast datasets (Elnaggar *et al.* 2021, Iuchi *et al.* 2021, Rao *et al.* 2021, Rives *et al.* 2021, Wittmann *et al.* 2021).

This contribution differs from previous efforts in several ways. First, it takes advantage of recent experiments that edited genotypes and measured fitness *in vivo* for more than 10^5^ DNA sequences (Papkou *et al.* 2023), a scale at which fitness prediction by deep learning becomes attractive. Specifically, I analyze *E.coli* fitness data on the antibiotic trimethoprim for almost 4^9^ ≈ 260 000 *E.coli* genotypes that differ at nine consecutive base pairs of the gene for dihydrofolate reductase (DHFR), which can convey trimethoprim resistance. (Papkou *et al.* 2023). For each amino acid sequence variant, the data comprises fitness measurements for nearly all synonymous DNA sequences. This is important, because sampling only some synonymous sequences is central for strategies to sample genotypes for experimental fitness measurements. Also, the data is nearly combinatorially complete on the nucleotide level, i.e. for variants at any two nucleotide sites, fitness data is also available for all combinations of these variants. This is important, because it helps to avoid sampling bias caused by combinatorially incomplete data.

Second and most importantly, I study how the quality of deep-learning based fitness predictions depends on how the training data is sampled. I show that random sampling and sampling of few synonymous DNA sequences per amino acid sequences leads to the best generalization performance on test data. In contrast, sampling maximally diverse nucleotide or amino acid sequences leads to the poorest performance. I show that these observations do not depend on the specific neural network architecture used, and are thus probably a property of the landscape itself.

## 2 Methods

### 2.1 Data

Unless otherwise mentioned, I use one-hot encoded DNA genotype data both for linear and nonlinear regression. To predict fitness for viable genotypes by (nonlinear) regression I used the 17 774 viable genotypes of the fitness data in (Papkou *et al.* 2023). This experimentally measured fitness data is a logarithmically transformed *E.coli* growth rate relative to a wild-type, which has a fitness of zero. It ranges between −1.17 and +1.4. All genotypes with fitness below −0.5 are inviable (Papkou *et al.* 2023). To avoid divergence of the mean absolute percentage error (mape) for fitness values around zero, I added an offset of +2 to all fitness values before training, so that they range between 0.83 and 3.4 after this transformation.

### 2.2 Neural network training

I trained neural networks of all architectures with the minibatch gradient descent method, using a batch size of 128 genotypes (Bertsekas 1996). To this end, I employed the widely used root mean square propagation (rmsprop) algorithm, as implemented in keras (tensorflow version 2.12.0, https://github.com/tensorflow/tensorflow/releases) (Chollet 2021) I tuned hyperparameters with a hyperband tuner implemented in tensorflow (version 2.12.0, tuner parameters: factor = 3, hyperband_iterations = 3) (Li *et al.* 2017). I used this hypertuner for 10 epochs per network, but stopped training for any one network when training showed no further improvement in performance for 5 epochs (Chollet 2021). See Supplementary Methods for details on the network architectures and the tuned hyperparameters.

### 2.3 Genotype sampling

I also restricted genotype sampling to the 17 774 viable genotypes, which encode 1630 unique amino acid sequences. For random (uniform) sampling of genotypes, I first randomly shuffled all viable genotypes and set aside 50% (8887) of them as a test set, and the remainder for validation and training. I then sampled a fixed number of the remaining genotypes for training and validation. I varied this number between *S *=* *200 (1.1% of all data) and *S *=* *8000 (45%) to explore how prediction quality depends on *S*. Because many of the resulting training/validation datasets were small, I did not use hold-out validation, but applied 4-fold cross-validation, setting aside 75% of the sample for training and 25% for validation, and repeated this procedure four times with non-overlapping validation datasets for each replicate. I computed the training and validation loss (mean squared error, mse, of predicted fitness) after each epoch as an average across the four training runs.

For each training sample I trained each network with the rmsprop algorithm for a maximum of 100 epochs with batch sizes of 128 samples. I stopped the training early when the training loss (mse) did not decrease for five consecutive epochs. I trained each network in three independent replicates to estimate how much fitness predictions vary across such replicates. I chose independent test and training/validation datasets for each value of *S* and for each replicate. I used the same procedure also for the non-random sampling procedures described in the text (Supplementary Methods).

## 3 Results

### 3.1 Recurrent neural networks are best at predicting the fitness of viable genotypes

Just like for other proteins (Li *et al.* 2019, Wu *et al.* 2019, Wittmann *et al.* 2021), only a small minority of the genotypes (17 774, 6.8%) in the DHFR trimethoprim resistance landscape is viable (Papkou *et al.* 2023). I study the ability of 6 neural network architectures to distinguish viable from inviable genotypes (Supplementary Results 1) and to predict the fitness of these viable genotypes by (nonlinear) regression.

As one of two base-line reference models to predict fitness, I use a random predictor. This predictor uses fitness values that are randomly shuffled among genotypes. It performs poorly, predicting less than 0.01% of the variation in fitness (Table 1). My second base-line reference model is linear regression, which already performs vastly better than random prediction, halving the mean errors (mean absolute percentage error, mape = 15.65%, mean absolute error, mae = 0.33), and increasing the correlation coefficients to *r *=* *0.66 and *R*^2^ = 0.41. In other words, linear regression can explain 41% of the variation in the data.

**Table 1.** Performance of deep learning network architectures on regression of viable genotypes.

	*R* ^2^	mae^a^	mape^b^	mse^c^	*r* ^d^	Parameters (×10^3^)^e^
Random	0.000082	0.61	28.41	0.56	0.01	n/a
Linear regression	0.41	0.33	15.65	0.16	0.66	n/a
Perceptron	0.92 (124.4%)^f^	0.12	5.96	0.025	0.96	7.75
RNN	0.94 (129.3%)	0.098	4.48	0.018	0.96	28.19
Convolutional	0.91 (122.0%)	0.15	6.70	0.039	0.95	16.23
Transformer	0.83 (102.4%)	0.21	9.40	0.073	0.91	4.91
RNN (codons)^g^	0.96 (134.1%)	0.081	3.65	0.013	0.98	33.92
Transformer (codons)^g^	0.93 (126.8%)	0.16	6.80	0.042	0.96	128.24

a Mean absolute error.

b Mean absolute percentage error.

c Mean squared error, the loss function used for network training.

d Spearman’s rank correlation coefficient *r*.

e Number of parameters in the best performing architecture.

f Numbers in parentheses indicate percent improvement relative to linear regression.

g Architecture with codon-based positional embedding.

The first neural network architecture I study is the multilayer perceptron (Rosenblatt 1958, Gurney 1997, LeCun *et al.* 2015), in which I tuned the number of layers, the number of neurons per layer, weight regularization, layer dropout, and the learning rate (Supplementary Methods). It already leads to a massive further improvement over linear regression. For example, it reduces the mape by 61.9%–5.96%, and increases *R*^2^ by 124.4% to *R*^2^ = 0.92. (See Table 1 for the other performance measurements).

The second architecture is a bidirectional recurrent neural network (RNN) (Hochreiter and Schmidhuber 1997), in which I tuned the number of bidirectional layers, the number of neurons in each layer, weight regularization, recurrent dropout, and the learning rate. This network performed slightly better than the perceptron, with *R*^2^ = 0.94 (129.3% improvement over linear regression) and a mape of 4.48%.

The third architecture is a one-dimensional convolutional network (LeCun *et al.* 2015), in which I tuned the number of convolutional layers, the number of dense layers that followed them, the number of neurons in these layers, their weight regularization, and the learning rate. It performed slightly less well (*R*^2^ = 0.91, 122% improvement over linear regression) than the preceding architectures.

The input to the three architectures I discussed thus far was a flattened one-hot encoded 9 × 4 = 36-dimensional representation of a DNA genotype. In contrast, the next architecture is a transformer (Vaswani *et al.* 2017), for which I first positionally embedded individual DNA sequences in a low-dimensional embedding space (Chollet 2021, p 347), which ensures that the embedding of each sequence also contains information about the position of each nucleotide in the sequence. The optimal embedding is learned during neural network training. I deliberately chose such end-to-end learning of word embedding, because it performs at a par with highly complex pretrained embeddings, may require lower embedding dimensions, and does not depend on other bioinformatic resources (Asgari and Mofrad 2015, Alley *et al.* 2019, Raimondi *et al.* 2019, ElAbd *et al.* 2020, Elnaggar *et al.* 2021, Iuchi *et al.* 2021, Rao *et al.* 2021, Rives *et al.* 2021).

In this transformer architecture, I tuned the number of embedding dimensions, the number of attention heads per transformer module, the size of each attention head, the number of neurons in each dense layer of a module, the number of stacked transformer modules, the dropout rate, and the learning rate (Supplementary Methods). Despite such extensive hypertuning, the transformer too performed less well than the RNN (*R*^2^ = 0.83, Table 1).

Feature engineering, i.e. choosing an appropriate representation of input data, can be crucial to improve network performance (Chollet 2021). For two further neutral networks, I chose a simple and general form of feature engineering with the advantage that it would apply to all protein-coding genes and is not specific to DHFR or a specific protein class. Specifically, I subdivided the 9 nucleotide input sequence into 3 integer-encoded codons and positionally embedded these codons into a space whose dimensionality I varied during hypertuning (Supplementary Methods). These codons became the input to a bidirectional RNN whose hyperparameters I also tuned (Supplementary Methods).

Feature engineering improves the performance of the transformer by a further 12.0% to *R*^2^ = 0.93), as well as that of the RNN by 2.1% to *R*^2^ = 0.96 (Table 1). Overall, the bidirectional RNN network (Supplementary Fig. S12b) with a codon-based embedding performs best, explaining 96% of the variation in fitness (mae = 0.081, mape = 3.65).

With 33 921 parameters the bidirectional RNN is more complex than the simpler and almost equally well-performing multilayer perceptron (7745 parameters, Table 1, Supplementary Results 2). The best-performing transformer requires many more parameters (128 241) despite its poorer performance. I focused my subsequent analyses on the best-performing RNN, but also compared their outcome with the best-performing perceptron and transformer, because of their widely varying complexity, to find out how strongly the influence of genotype sampling on prediction performance depends on the architecture. During training, all three types of networks converge rapidly (within 10 epochs) to their optimal performance (Supplementary Fig. S1). They show no signs of overfitting thereafter, (Supplementary Fig. S1), suggesting that even better architectures exist.

### 3.2 A Small sample of training data can suffice to predict fitness with high accuracy

Because measuring fitness experimentally is laborious, any training sample of genotypes with measured fitness should be as small as possible. This is especially important when high-throughput fitness measurements are infeasible (Wittmann *et al.* 2021, Nikolados *et al.* 2022). To find out whether accurate fitness prediction is even possible from a small sample, I first studied how the prediction quality of the best-performing RNN varies with the sample size *S* that is used for training and validation. Specifically, I varied this sample size between *S *=* *200 and *S *=* *8000 randomly chosen genotypes (1.1%–45.0% of all viable genotypes). For any one value of *S*, I subdivided all 17 774 viable genotypes into a test set that comprised 50% of the data (8886 sequences), and a set for training and validation that comprised *S* sequences, using 4-fold cross-validation during training. Subsequently, I tested the model thus trained on the test set.

**Figure 1.**
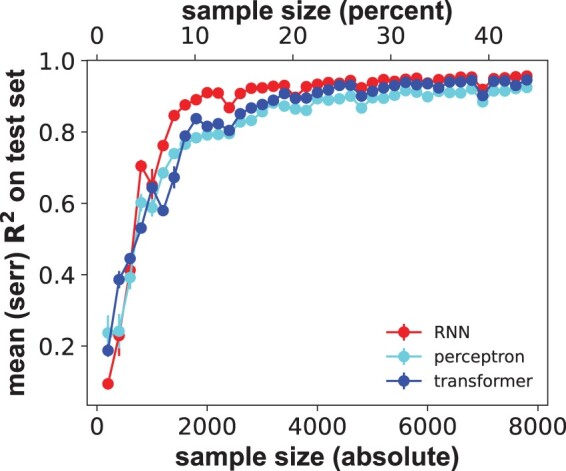
Predictive performance increases rapidly with sample size for three different neural network architectures. Horizontal axis: sample size *S* of a random genotype sample used for training and validation through 4-fold cross-validation, both in absolute numbers of genotypes (bottom) and as a percentage of all viable genotypes (top). Vertical axis: performance of the three major network architectures (legend) on a test-set comprising 50% (8887) of viable genotypes, as quantified by the coefficient of determination *R*^2^, between measured and predicted fitness. Whiskers indicate one standard error of the mean based on three replicate trainings for each network and sample size.

Figure 1 shows the coefficient of determination *R*^2^ of predicted fitness on the test set as a function of sample size for the best-performing RNN (Supplementary Fig. S12b), and for random (uniform) sampling of the training data. *R*^2^ increases rapidly with sample size *S*, and reaches 90% of the *R*^2^ obtained for the maximal sample size after training on only 7.8% (1400) of genotypes. Notably, the sample sizes needed to reach a value of *R*^2^ within 90% of that for the largest training set are similarly small for the multilayer perceptron and for the transformer (1600 genotypes, 9.0% of all genotypes for both, Fig. 1). Other measures of performance also reach close to peak performance with small samples (Supplementary Fig. S2). In sum, accurate fitness prediction is possible with small training sets, independently of network architecture.

### 3.3 Sampling strategies that reduce the number of synonymous sequences alter performance only slightly

Random (uniform) samples of DNA sequences for fitness measurements have a key disadvantage. Because of the redundancy of the genetic code, many sampled DNA sequences will be synonymous, encoding the same amino acid sequences. Because fitness differences between synonymous sequences are usually much smaller than between non-synonymous sequences, laborious fitness measurements for synonymous sequences can waste valuable experimental resources (McDonald and Kreitman 1991, Cuevas *et al.* 2011, Bailey *et al.* 2021).

These observations raise the question how much predictive power a deep learning network loses when sampling few or no synonymous sequences for each amino acid sequence. To answer this question, I first implemented a sampling procedure (“one syn.”) that aims to create training/validation datasets in which every amino acid is only represented by a single nucleotide sequence, thus avoiding synonymous sequences altogether. Because all viable 17 774 DNA sequences encode only 1630 amino acid sequences, synonymous sequences can only be avoided entirely for small samples (Supplementary Methods). However, the procedure creates a mean number of nucleotide sequences per amino acid sequences that is much smaller than for random samples [e.g. *S *=* *1400: “one syn.” creates 1.06 ± 0.004 (mean ± 1 standard error) synonymous sequences per amino acid sequences; random: 2.04 ± 0.03 synonymous sequences].

**Figure 2.**
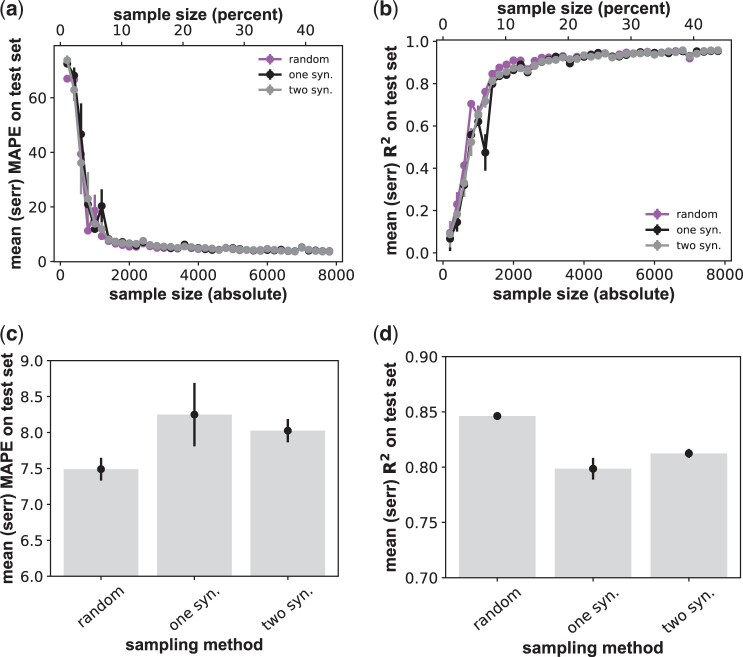
Sampling one or two synonymous sequences moderately degrades RNN prediction quality. (a) Horizontal axis: size *S* of the genotype sample used for training and validation through 4-fold cross-validation, both in absolute numbers of genotypes (bottom) and as a percentage of all viable genotypes (top). Vertical axis: prediction quality of the (best-performing) RNN architecture, as quantified by the mape of fitness prediction as a function of sample size *S*. The *S* genotypes are either sampled randomly and uniformly (“random”), or such that only one synonymous (“one syn.”) or two synonymous (“two syn.”) nucleotide sequences are sampled per amino acid sequence (Supplementary Methods). Whiskers indicate one standard error of the mean, based on three replicate trainings for each network and sample size. (b) like (a), but prediction quality is quantified through the coefficient of determination *R*^2^. (c) Dot-whisker plot indicating the means (height of bars) and standard errors (whiskers) of the mape at a fixed sample size of *S *=* *1400 genotypes for the three sampling methods shown on the horizontal axis. (d) like (c), but for *R*^2^ instead of the mape.

I hypothesized that this sampling method leads to better predictions than random sampling, because it samples the most informative nucleotide sequences, i.e. those that encode different proteins. However, this is not the case (Fig. 2a and b). Here and below, I compare sampling performance mostly at *S *=* *1400, because this is where the RNN first reaches 90% of its peak performance, i.e. its performance for the largest training sample. This is also where different architectures show the clearest performance differences (Fig. 1). At this sample size, the mape of the RNN increases by 10% (to 8.25 ± 0.44) for “one syn.” sampling relative to random sampling (7.49 ± 0.16, Fig. 2c), and the mean *R^2^* decreases by 5.9% (to 0.80 from 0.85, Fig. 2d). Likewise, this sampling method does not lead to a consistent and substantial performance improvement for the other network architectures (Supplementary Figs. S3 and S4; multilayer perceptron: mape = 9.8 ± 0.24 and 9.53 ± 0.09; *R*^2^ = 0.74–0.71; transformer: mape = 11.1 ± 0.92 and 11.9 ± 0.29; *R*^2^ = 0.67 and 0.63, each pair of numbers for random sampling and “one syn.” respectively).

A next, less extreme sampling method (“two syn.”) aims to create samples where every amino acid sequence is encoded by two randomly chosen nucleotide sequence. The exception is amino acid sequences that are presented by only a single encoding nucleotide sequence in the data, and large samples, where the smallest number of nucleotide sequences beyond two is sampled per amino acid sequence (Supplementary Methods). The rationale for this procedure is that it may be necessary to capture at least some of the diversity of synonymous sequences to predict fitness most accurately. (Ideally, one would sample synonymous sequences that differ in fitness, but this is not possible, because genotype fitness is unknown at the time of sampling.)

This method performs similar to “one syn.” sampling (Fig. 2). Specifically, at *S *=* *1400 the RNN’s mape is 8.03 ± 0.16, as compared to 8.25 ± 0.44 for “one syn.,” and its *R*^2^ equals 0.81 (one syn.: 0.80). The method also leads to similar performance for the other two network architectures (Supplementary Figs. S3 and S4; multilayer perceptron: mape = 9.53 ± 0.09 and 10.4 ± 0.18; *R*^2^ = 0.71 and 0.67; transformer: mape = 11.9 ± 0.29 and 10.5 ± 0.43; *R*^2^ = 0.63 and 0.69, each pair of numbers for “one syn.” and “two syn.,” respectively).

In sum, independent of the neural network architecture, genotype sampling of few synonymous sequences does not dramatically alter performance relative to random sampling. Other methods for codon compression (Pines *et al.* 2015), i.e. reducing synonymous sampling, are discussed in Supplementary Results 3.

### 3.4 Increasing sampled sequence diversity reduces predictive performance substantially

In a random (uniform) sample of DNA nucleotide sequences, some sequences may be very similar to one another. Such sequences tend to encode amino acid sequences that are identical or at least physicochemically similar, and may thus have similar fitness (Freeland and Hurst 1998). It may be best to avoid such highly similar sequences during neural network training, and instead sample more diverse sequence to facilitate generalization to a test dataset.

**Figure 3.**
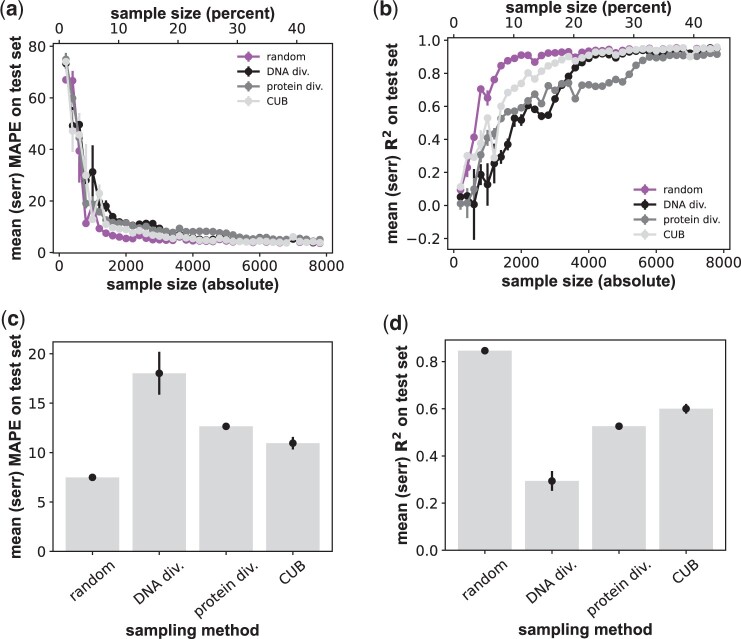
Sampling for sequence diversity or preferred codon usage substantially reduces RNN prediction quality. (a) Horizontal axis: size *S* of the genotype sample used for training and validation through 4-fold cross-validation, both in absolute numbers of genotypes (bottom) and as a percentage of all viable genotypes (top). Vertical axis: prediction quality of the (best-performing) RNN architecture, as quantified by the mape of fitness prediction as a function of sample size *S*. The *S* genotypes are either sampled randomly and uniformly (“random”), to achieve maximal DNA sequence diversity (“DNA div.”), maximal amino acid sequence diversity (“protein dev.”), or maximal codon usage bias (“CUB,” Supplementary Methods). Whiskers indicate one standard error based on three replicate trainings for each network and sample size. (b) like (a), but prediction quality is quantified through the coefficient of determination *R*^2^. (c) Dot-whisker plot indicating the means (height of bars) and standard errors (whiskers) of the mape at a fixed sample size of *S *=* *1400 genotypes for the three sampling methods shown on the horizontal axis. (d) like (c), but for *R*^2^ instead of the mape.

I tested this hypothesis with two complementary sequence sampling procedures. The first aims to maximize nucleotide sequence diversity in a training/validation data sample (Supplementary Methods). Remarkably, this procedure performs substantially worse than random sampling (Fig. 3). At a sample size of *S *=* *1400 sequences, the mape of the RNN increases by 140.6% to 18.02 ± 2.2 (Fig. 3c), relative to random sampling (7.49 ± 0.16). The mean *R*^2^ decreases by 66.9% (from 0.85 to 0.29, Fig. 3d). This sampling method also degrades the performance of the other network architectures to a similar extent (Supplementary Figs. S8 and S9).

My second procedure aims to sample a set of amino acid sequences whose constituent sequences are physicochemically maximally diverse (Supplementary Methods), using a high-dimensional representation of each amino acid (Georgiev 2009) that outperforms others in similar machine learning tasks (Wittmann *et al.* 2021). This method too substantially degrades prediction quality relative to random sampling. For the RNN at a sample size of *S *=* *1400, it increases the mape by 70% from 7.49 ± 0.16 to 12.7 ± 0.19 (Fig. 3c). It decreases the *R^2^* by 37.6% from 0.85 to 0.53 (Fig. 3d). Performance also declines to a similar extent for the other two architectures (Supplementary Figs. S8 and S9).

### 3.5 Sampling sequences with high codon usage bias

I next studied a sampling procedure that preferentially samples nucleotide sequences with high codon usage bias (Supplementary Methods). Such sequences often encode proteins that are highly expressed, hence more easily studied, and thus preferred for experimental analysis (Ikemura 1985, Hershberg and Petrov 2008, Komar 2016, Iriarte *et al.* 2021). This procedure degrades prediction quality relative to random sampling, but more modestly than diversity-maximizing sampling. Specifically, for the RNN at a sample size of *S *=* *1400, it increases the mape by 46.9% from 7.49 ± 0.16 to 11.0 ± 0.63 (Fig. 3c), and decreases the *R*^2^ by 29.4% from 0.85 to 0.6 (Fig. 3d, Supplementary Figs. S8 and S9; perceptron: mape increases by 26.5% from 9.8 ± 0.24 to 12.4 ± 0.77; *R*^2^ decreases by 27.0% from 0.74 to 0.54; transformer: mape increases by 15.3% from 11.1 ± 0.92 to 12.8 ± 0.28; *R*^2^ decreases by 20.9% from 0.67 to 0.53, all numbers for *S *=* *1400). The performance differences between the sampling methods I studied also persist at much larger sample sizes, albeit at much smaller absolute performance differences (Supplementary Fig. S10).

## 4 Discussion

Random sampling leads to the best generalization of fitness predictions, followed by sampling few synonymous DNA sequences per amino acid sequence. The latter observation is easily explained by the weak fitness effects of synonymous mutations (McDonald and Kreitman 1991, Cuevas *et al.* 2011, Bailey *et al.* 2021), which means that synonymous DNA sequences account for less fitness variation than non-synonymous sequences.

In contrast to random sampling, sampling genotypes for highly diverse DNA sequences or highly physicochemically diverse amino acid sequences substantially degrades generalization ability. Such sampling for diversity disfavors sequences within local neighborhoods. Random sampling from a small sequence space like the one I study here will cause at least some sampled sequences to lie close to each other. My observations show that such highly local sampling is important for accurate fitness predictions. This observation is consistent with theoretical work that examined the ability of quadratic regression models to predict the fitness of RNA molecules, as determined by a biophysically motivated algorithm for RNA secondary structure folding (du Plessis *et al.* 2016).

These observations hold not just for the (best-performing) RNN, but also for perceptrons and transformers. They are thus probably a property of the landscape and the sampling regime rather than of a specific neural network architecture. I also found that 90% of the peak performance for larger training samples can be reached with a sample of merely 1400–1600 viable sequences (<10% of all viable sequences). This is consistent with previous observations of successful phenotype prediction from small training samples of 10^1^–10^3^ genotypes for other machine learning methods (Wittmann *et al.* 2021, Nikolados *et al.* 2022).

A recent study examined the role of sampled sequence diversity to predict the translation efficiency of a bacterial fluorescent reporter gene with deep learning models (Nikolados *et al.* 2022). The 200 000 96 nt sequences in this study were organized around 56 seed sequences that are distant from each other in the large space of 4^96^≈6 × 10^57^ DNA sequences of this length (Cambray *et al.* 2018). Each of these seed sequences was mutagenized to create a local “cloud” of ≈4000 sequences around the seed whose translation efficiency was measured. The study showed that training a deep learning neural network only on the sequences near one seed yields poor generalization for test data derived from sequences far from the seed. Performance substantially improved when data from an increasing number of seeds was used in training, even if the total number of sequences in the training data was held constant (Nikolados *et al.* 2022).

The apparent discrepancy to my observation that sampling for sequence diversity leads to poor generalization can be easily explained by the smaller region of sequence space I sample. In much larger sequence spaces, sampling for diverse sequences may become essential to ensure generalization to unseen sequences. Finding the optimal balance between “global” sampling of distant sequences and “local” sampling around these distant sequences remains an important task for future work.

In addition to sampling diverse genotypes, sampling genotypes for favorable codon usage also substantially degrades generalization ability. One candidate explanation is that such sampling may reduce the variation of fitness in a sample, because it reduces expression variation as a contributor to fitness variation. However, this is not the case, because genotype samples with high codon usage do not vary less in fitness than random samples (e.g., fitness standard deviation (SD) in three samples of *S *=* *1400 genotypes: SD = 0.56 ± 0.002 when sampling for high codon usage bias, and SD = 0.53 ± 0.007 for a random sample). To explain why training samples with high codon usage bias leads to low generalization ability remains another task for future work.

For neural network training, fitness data can in principle be integrated with other pertinent information, e.g. about protein expression or protein structure. Doing so would either require high throughput measurements of expression and structure for thousands of protein variants, or reliable computational predictions thereof. Whether such additional information may affect the sampling behavior of fitness predictions, and lead to smaller required sample sizes also remain questions for future work.

The small sequence space of the experimental fitness landscape I study is one main limitation of my work. Another is that I study only one landscape, because it is the only one currently available with fitness data for most synonymous genotypes encoding an amino acid sequence. Other landscapes may require different kinds of sampling regimes. For example, a landscape of mRNAs translational efficiency is affected by multiple and heterogeneous factors, including mRNA secondary structure and hydrophobicity of the encoded peptide (Cambray *et al.* 2018). Such a landscape may thus require more diverse sampling than the landscape of an enzyme’s catalytic activity. Until many and diverse landscapes have been studied, simple sampling regimes like random sampling or codon compression sampling will be the best starting points to train deep learning neural networks on experimentally mapped fitness landscapes.

## Supplementary Material

btae317_Supplementary_Data
